# MicroRNA 452 Regulates Cell Proliferation, Cell Migration, and Angiogenesis in Colorectal Cancer by Suppressing VEGFA Expression

**DOI:** 10.3390/cancers11101613

**Published:** 2019-10-22

**Authors:** Ji Su Mo, Won Cheol Park, Suck-Chei Choi, Ki Jung Yun, Soo-Cheon Chae

**Affiliations:** 1 Department of Pathology, School of Medicine, Wonkwang University, Iksan, Chonbuk 54538, Korea; siuale97@hanmail.net (J.S.M.); kjyun@wku.ac.kr (K.J.Y.); 2 Digestive Disease Research Institute, Wonkwang University, Iksan, Chonbuk 54538, Korea; parkwc@wku.ac.kr (W.C.P.); medcsc@wku.ac.kr (S.-C.C.)

**Keywords:** microRNA, MIR452, VEGFA, VEGFR2, angiogenesis, colorectal cancer

## Abstract

The human microRNA 452 (*MIR452*) was identified as a colorectal cancer (CRC)-associated micro RNA (miRNA) by miRNA expression profiling of human CRC tissues versus normal colorectal tissues. It was significantly up-regulated in human CRC tissues. However, the functional mechanisms of *MIR452* and its target genes in CRC remain unclear. We identified 27 putative *MIR452* target genes, and found that the vascular endothelial growth factor A (*VEGFA*) was a direct target gene of *MIR452*. Both cellular and extracellular VEGFA levels were significantly downregulated in CRC cells upon their transfection with *MIR452* or *siVEGFA*. VEGFA expression was frequently downregulated in human CRC tissues in comparison with that in their healthy counterparts. We showed that MIR452 regulated the expression of genes in the VEGFA-mediated signal transduction pathways vascular endothelial growth factor receptor 1 (VEGFR2)–mitogen-activated protein kinase (MAPK) and VEGFR2–SRC proto-oncogene non-receptor tyrosine kinase (SRC) in CRC cells. Immunohistological analyses of xenografted *MIR452*-overexpressing CRC cells in mice showed that *MIR452* regulated cell proliferation and angiogenesis. Furthermore, aortic ring angiogenesis assay in rats clearly showed that the number of microvessels formed was significantly reduced by *MIR452* transfection. Our findings suggest that *MIR452* regulates cell proliferation, cell migration, and angiogenesis by suppressing VEGFA expression in early CRC progression; therefore, *MIR452* may have therapeutic value in relation to human CRC.

## 1. Introduction

Colorectal cancer (CRC) is the third most prevalent type of cancer worldwide [1]. The cause of CRC is multifactorial, including genetic variation and epigenetic and environmental factors such as diet, microbiome, and their metabolites [2]. However, the precise molecular mechanism underlying the development and progression of CRC remains largely unknown. Therefore, it is of great importance to elucidate the molecular mechanisms and genes underlying CRC tumorigenesis.

MicroRNAs (miRNAs) are endogenously expressed small noncoding RNA molecules that mostly bind to the 3′ untranslated regions (UTR) of their target mRNAs, thereby regulating gene expression in multicellular organisms post-transcriptionally by controlling the stability or translation of target mRNAs [3]. MiRNAs regulate crucial biological processes, such as cell proliferation, apoptosis, and differentiation as well as angiogenesis [4,5,6,7]. They also affect the pathogenesis of various cancer types by functioning as oncogenes or tumor suppressor genes [8,9,10,11]. More recently, accumulating reports suggest that miRNAs are associated the various forms of tumorigenesis including lung cancer [12,13,14], prostate cancer [15,16], gastric cancer [17,18], and liver cancer [19,20]. Therefore, characterization of the correlations between miRNAs and their target genes in cancer cells may have a substantial diagnostic, prognostic, and therapeutic value.

MicroRNA 452 (*MIR452*, also known as has-miR-452) is encoded by the chromosomal region Xq28 in humans and clustered together with *MIR224* within the gamma-aminobutyric acid A receptor epsilon subunit (*GABRE*) gene. Several studies have suggested that *MIR452* expression is downregulated in human breast cancer [21], glioma [22], and hepatocellular carcinoma (HCC) [23]. However, in other studies, *MIR452* has been found to be upregulated in hepatocellular carcinomas [24] and lymph node-positive urothelial carcinomas [25], suggesting that *MIR452* can have diverse roles in distinct types of human cancers or cells. In our previous study, *MIR452* was found to be upregulated in both CRC [26] and colitis [27] by differential miRNA expression profiling of human CRC tissues and dextran sulfate sodium (DSS)-induced mouse colitis tissues, respectively.

In this study, we identified the target genes of *MIR452* and analyzed their functions in human CRC cell lines and colorectal tissues. The candidate *MIR452* target genes were identified by microarray-based differential mRNA expression profiling of *MIR452*-overexpressing CRC cells. These candidates were shortlisted by comparing the mRNA microarray results for the candidate target genes predicted by bioinformatics tools. We identified vascular endothelial growth factor A (*VEGFA*, also known as *VPF, VEGF*, or *MVCD1*) as a *MIR452* target gene in CRC, and analyzed the correlation between *MIR452* and *VEGFA* using human CRC tissues, cell lines, and xenograft tumors, as well as rat aortas. Overall, we demonstrated that *MIR452* regulates cell proliferation and migration as well as angiogenesis in CRC by suppressing VEGFA expression.

## 2. Results

### 2.1. MIR452 Expression Level in Human CRC Tissues and Cell Lines

We have previously found that *MIR452* is upregulated in human CRC tissues [26]. To confirm this result, we compared the *MIR452* levels in 10 human CRC tissue samples with those in matching healthy colon tissues by qRT-PCR. *MIR452* levels were increased in CRC tissues (7 out of 10, Figure S1A). To determine the levels of endogenous MIR452 in CRC cell lines such as SW480, HT29, Caco2, HCT116, Lovo, and SW48 cells, we carried out qRT-PCR analysis using the total RNAs isolated from each cell lines. The MIR452 level was lowest in SW480 cells and highest in HT29 cells (Figure S1B).

### 2.2. Differential mRNA Expression Profiling of MIR452-Overexpressing Cells

To identify the genes downregulated by *MIR452* overexpression, a *MIR452* mimic was transfected into SW480 and Caco2 cells. Increased *MIR452* level 24 h after the transfection confirmed the transfection efficiency (Figure S1C). The cells were harvested 48 h after the transfection for mRNA expression profiling with the Illumina HumanHT-12 v4 Expression BeadChip. We identified 261 genes whose levels were 1.3-fold downregulated in *MIR452*-overexpressing cells (Table S1).

### 2.3. Identification of the MIR452 Target Genes

The 261 genes identified by mRNA microarray analysis in *MIR452*-overexpressing cells were compared with the candidate *MIR452* target genes predicted by TargetScan and miRWalk algorithms. Of the 261 genes, 27 genes were finally identified as putative direct targets of *MIR452* (Table 1). Among them, we focused on *VEGFA*. We observed that SW480 and SW48 cells had the lowest and highest endogenous *VEGFA* levels, respectively (Figure S1D).

### 2.4. VEGFA Was a Direct Target of MIR452

**Figure 1:**
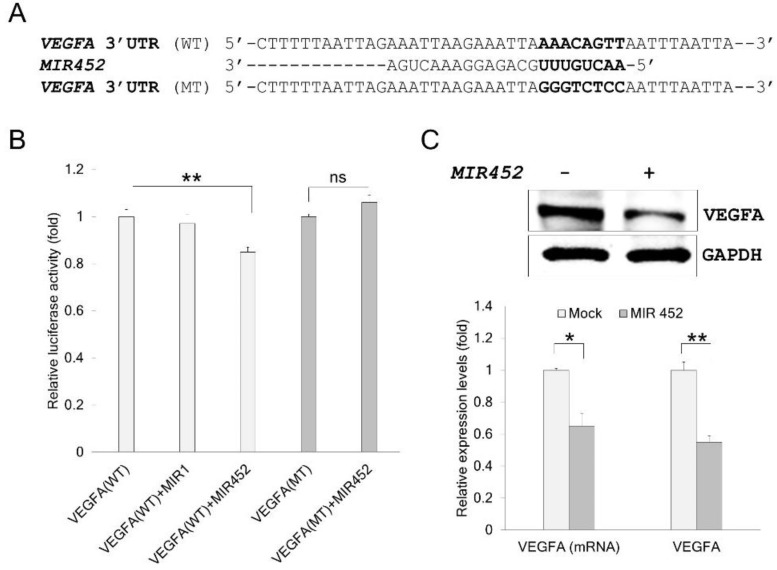
*VEGFA* was a direct target of *MIR452*. (**A**) Sequence alignment of the wild type (WT) and mutated (MT) *MIR452* target sites in the 3′-UTR of *VEGFA*. A human *VEGFA* 3′-UTR containing the WT and MT *MIR452* binding site was cloned downstream of a luciferase reporter gene. (**B**) The luciferase reporter plasmid containing the WT or MT *VEGFA* 3′-UTR was co-transfected into Caco2 or HT29 cells with the *MIR1* mimic (negative control) or *MIR452* mimic. Luciferase activity was determined by the dual luciferase assay. Results are shown as the relative firefly luciferase activity normalized to the Renilla luciferase activity. Data represent three independent experiments with the Caco2 cells. (**C**) *VEGFA* mRNA and protein levels in the mock- and *MIR452* mimic-transfected Caco2 cells. Protein or mRNA was extracted 72 or 48 h after transfection, respectively, and the samples were subjected to western blotting or qRT-PCR. Data represent three independent experiments. Statistical differences were calculated using Student’s *t*-test (ns = not significant; * *p* < 0.05; ** *p* < 0.01).

To assess whether *MIR452* directly interacts with *VEGFA* 3′-UTR, we cloned wild type (WT) *VEGFA* 3′-UTR (predicted to interact with *MIR452*) into a luciferase reporter vector (Figure 1A). The luciferase intensity was reduced by approximately 15% when the cells were co-transfected with a *MIR452* mimic (*p <* 0.01, Figure 1B). As a negative control, a *MIR1* mimic instead of the *MIR452* mimic was co-transfected with the wild WT *VEGFA* 3′-UTR construct. The *MIR1* mimic did not affect the luciferase activity (Figure 1B). As an additional negative control, we also cloned a mutated (MT) version of *VEGFA* 3′-UTR whose eight of the bases complementary to *MIR452* were substituted (Figure 1A). As expected, the luciferase activity did not change (Figure 1B). Next, we tested whether *MIR452* regulated *VEGFA* mRNA and protein levels in Caco2 cells. The *VEGFA* mRNA level was lower in Caco2 cells transfected with the *MIR452* mimic than in un-transfected control cells (*p* < 0.05; Figure 1C). The cellular VEGFA protein levels were also significantly reduced in the *MIR452*-overexpressing cells (*p* < 0.01; Figure 1C). These results indicate that *VEGFA* is a direct target of *MIR452*.

### 2.5. VEGFA Expression in Human CRC Tissues

**Figure 2:**
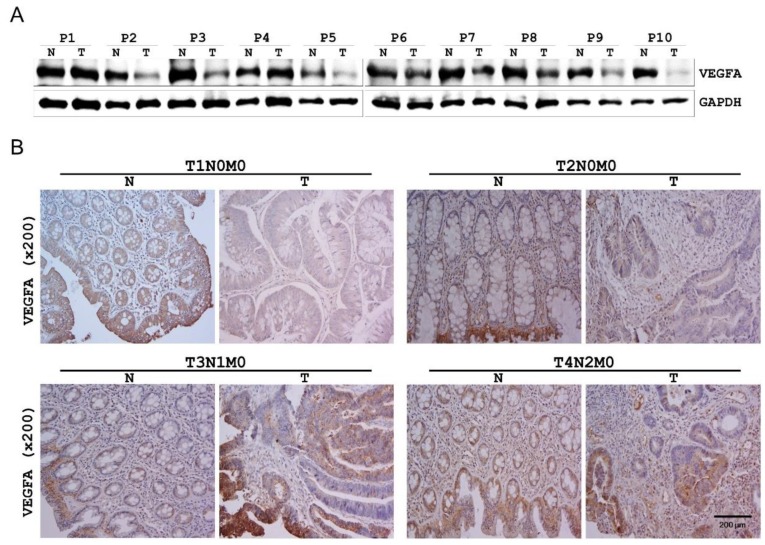
Endogenous VEGFA levels in human colorectal cancer (CRC) tissues. (**A**) The expression levels of VEGFA were validated using 10 pairs of human CRC and adjacent healthy colorectal samples by western blotting. The expression levels were normalized to that of Glyceraldehyde 3-phosphate dehydrogenase (GAPDH). (**B**) VEGFA immunostaining in the TNM Classification of Malignant Tumors (TNM) stage human CRC and adjacent healthy colorectal samples (200× magnification). These experiments were independently performed three times in duplicate.

On the basis of the findings described above, we evaluated VEGFA expression in additional 10 human CRC tissues and matching healthy colon tissues by western blotting. VEGFA protein expression was found to be decreased in CRC tissues in 8 of the 10 pairs (Figure 2A).

The finding described above (Figure S1A and Figure 2A) prompted us to evaluate VEGFA expression in different tumor (T) stages of human CRC and the matching healthy colon tissues by immunohistochemistry. VEGFA level was significantly lower in the T1 and T2 stage CRC tissues relative to that in the matching healthy colon tissues. However, VEGFA level dramatically increased in the T3 stage CRC tissue and no difference was observed at the T4 stage (Figure 2B).

### 2.6. MIR452 Regulated the VEGFA–Vascular Endothelial Growth Factor Receptor (VEGFR) Signaling Pathway in CRC Cells

**Figure 3:**
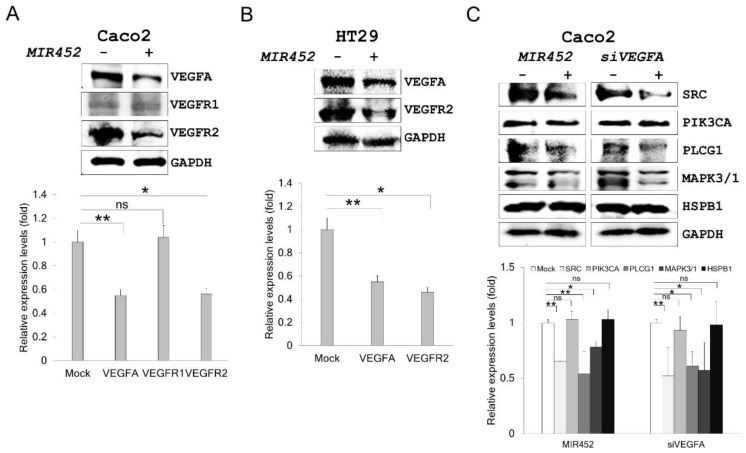
Expression levels of the target and downstream genes in *MIR452*-overexpressing CRC cells. (**A**) The expression levels of VEGFR1 and VEGFR2 in Caco2 cells upon *MIR452* overexpression. (**B**) The VEGFR1 and VEGFR2 expression levels in *MIR452*-overexpressing HT29 cells. (**C**) Proto-oncogene non-receptor tyrosine kinase (SRC), phosphatidylinositol-4,5-bisphosphate 3-kinase catalytic subunit alpha (PIK3CA), phospholipase C gamma 1 (PLCG1), mitogen-activated protein kinase (MAPK), and heat shock protein family B member 1 (HSPB1) expression levels in Caco2 cells transfected with the *MIR452* mimic or *VEGFA* gene silencing (*siVEGFA*). Three independent experiments were performed with duplicates, and the statistical differences were calculated using Student’s *t*-test (ns = not significant; * *p* < 0.05; ** *p* < 0.01).

We then determined which VEGFA receptor is mainly regulated by *MIR452* using *MIR452*-transfected Caco2 cells. VEGFR2 (*p* < 0.05) was more significantly affected by *MIR452* overexpression than VEGFR1 (Figure 3A). Downregulation of VEGFR2 by *MIR452* overexpression was also validated in HT29 cells (*p* < 0.05; Figure 3B).

To determine the functional significance of the interaction between *MIR452* and VEGFR2 in Caco2 cells, we analyzed the expression levels of several proteins involved in the signal transduction pathway downstream of VEGFR2 by western blot analysis. Proto-oncogene non-receptor tyrosine kinase (SRC), phospholipase C gamma 1 (PLCG1), and mitogen-activated protein kinase (MAPK) levels were significantly reduced by *MIR452* transfection in Caco2 cells (*p* < 0.01, *p* < 0.01, and *p* < 0.05, respectively; Figure 3C). However, phosphatidylinositol-4,5-bisphosphate 3-kinase catalytic subunit alpha (PIK3CA) and heat shock protein family B member 1 (HSPB1) levels did not change by *MIR452* transfection (Figure 3C). We also obtained similar results by knocking down *VEGFA* in Caco2 cells through *VEGFA* gene silencing (*siVEGFA*) transfection (Figure 3C). These results indicated that *MIR452* regulated the VEGFA–VEGFR2-mediated SRC, PLCG1, and MAPK signal transduction pathways, but not PIK3CA or HSPB1 signaling in CRC cells.

### 2.7. MIR452 Regulated VEGFA-Mediated VEGFR2–SRC–Protein Tyrosine Kinase 2 (PTK2) Signaling

**Figure 4:**
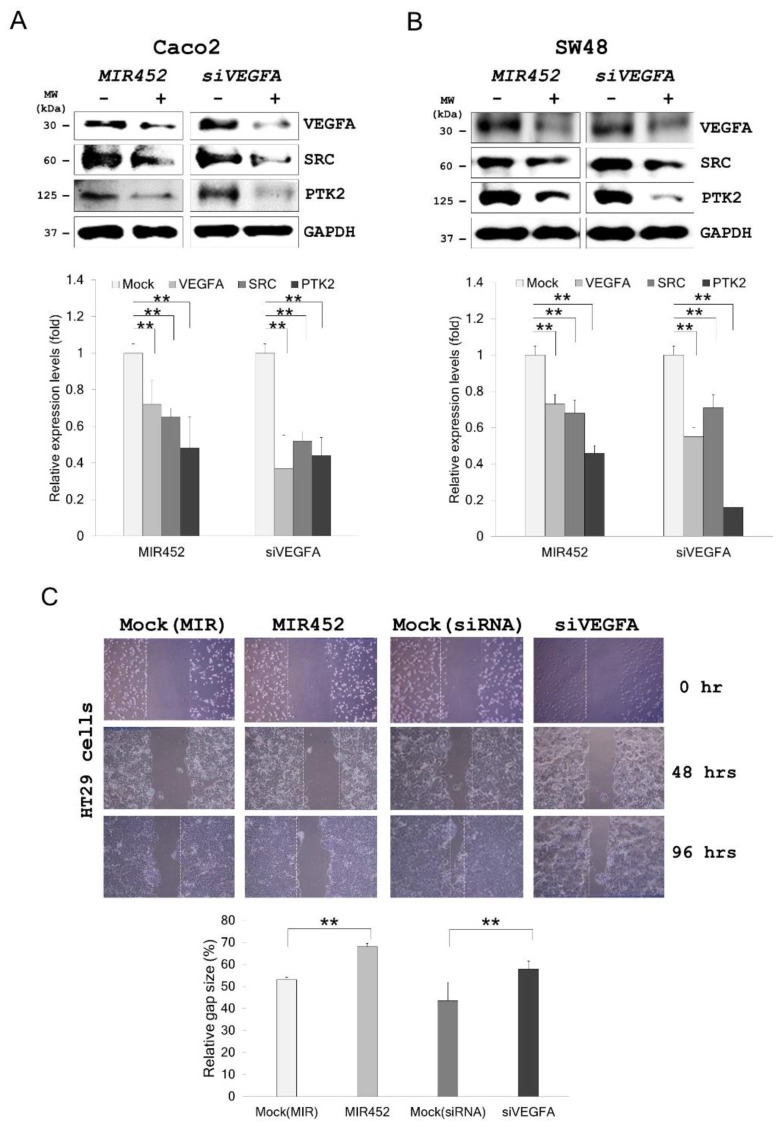

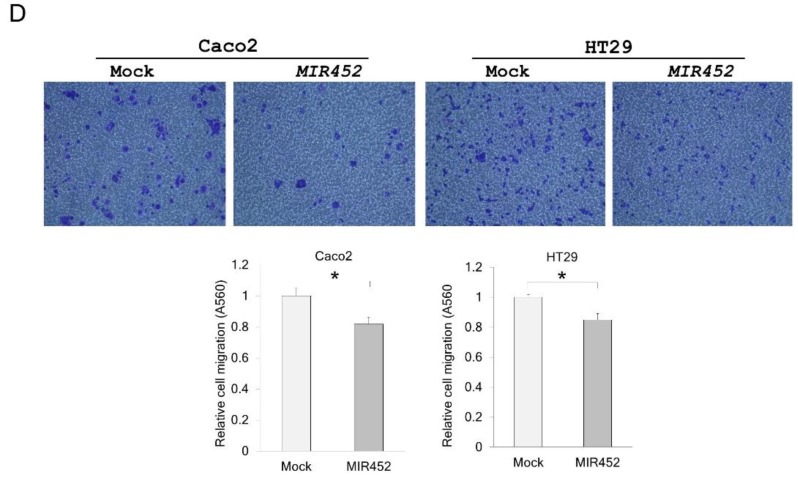
*MIR452* regulated VEGFA-mediated VEGFR2–SRC signaling in CRC cells. (**A**) Western blot analysis of VEGFR2-regulated proteins, SRC and protein tyrosine kinase 2 (PTK2), in Caco2 cells. (**B**) Western blot analysis of SRC and PTK2 in SW48 cells. Three independent experiments were performed in duplicate, and the *p*-values were calculated using Student’s *t*-test (** *p* < 0.01). (**C**) The scratch wound assay was conducted using HT29 cells transfected with the *MIR452* mimic, *siVEGFA*, or mock controls. The migration distance was measured 0, 48, and 96 h after the cells were scratched. Three independent experiments were performed in duplicate, and the *p*-values were calculated using Student’s *t*-test (** *p* < 0.01). (**D**) For the migration assay, we used 24-well Transwell chambers, which separated the upper and lower compartments by polycarbonate membranes that consisted of 8 μm pores. The cells that retained the dye were quantified by measuring absorbance at 560 nm (A560). Compared with the mock control, the *MIR452* mimic reduced migration of both Caco2 and HT29 cells. Three independent experiments were performed with duplicates, and the *p*-values were calculated using Student’s *t*-test (* *p* < 0.05).

On the basis of the above results, we tested whether VEGFA downregulation by the *MIR452* mimic or *siVEGFA* affected the VEGFR2–SRC–PTK2 signaling pathway in CRC cells. Western blotting results showed that SRC (*p* < 0.01) and PTK2 (*p* < 0.01) were markedly downregulated by both *MIR452* mimic and *siVEGFA* transfections in Caco2 and SW48 cells (Figure 4A,B). These findings suggested that *MIR452* regulated the VEGFA-mediated VEGFR2–SRC–PTK2 signaling pathway.

### 2.8. Migration of the Cells Transfected with the MIR452 Mimic or siVEGFA

As shown in Figure 4C, scratch wound assay showed that migration of HT29 cells was significantly suppressed upon transfection with the *MIR452* mimic or *siVEGFA* (Figure 4C, *p* < 0.01). Likewise, migration of Caco2 and HT29 cells through the Transwell filters was also significantly suppressed by the *MIR452* mimic (Figure 4D, *p* < 0.05). These results suggested that *MIR452* regulated cell migration by the VEGFA-mediated VEGFR2–SRC–PTK2 signaling pathway in CRC cells.

### 2.9. MIR452 Regulated VEGFA-Mediated VEGFR2–KRAS GTPase Proto-Oncogene (KRAS)–B-Raf Proto-Oncogene, Serine/Threonine Kinase (BRAF) Signaling

**Figure 5:**
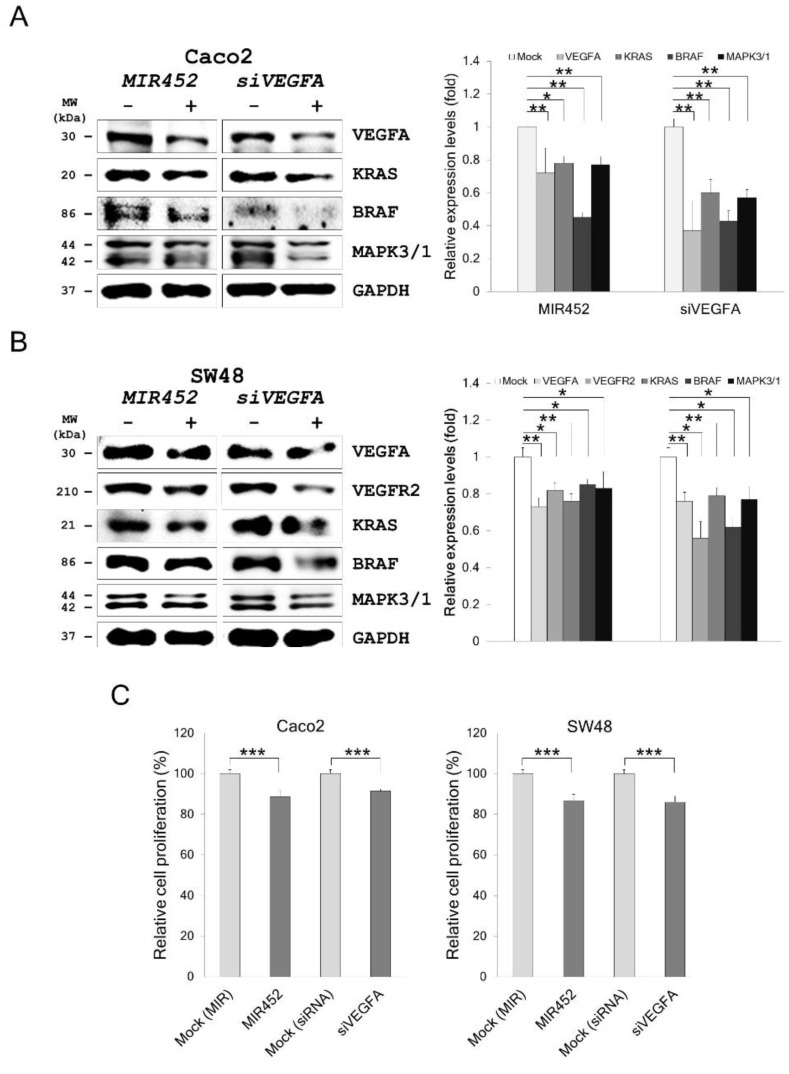
*MIR452* regulated VEGFA-mediated VEGFR2–KRAS GTPase proto-oncogene (KRAS) signaling in CRC cells. (**A**) Western blot analysis of VEGFR2-regulated proteins KRAS, B-Raf proto-oncogene, serine/threonine kinase (BRAF), and MAPK in Caco2 cells transfected with the *MIR452* mimic or *siVEGFA*. (**B**) Western blot analysis of KRAS, BRAF, and MAPK in SW48 cells transfected with the *MIR452* mimic or *siVEGFA*. Four independent experiments were performed in duplicate, and the *p*-values were calculated using Student’s *t*-test (* *p* < 0.05; ** *p* < 0.01). (**C**) Viability of Caco2 and SW48 cells transfected with the *MIR452* mimic or *siVEGFA*. Cell viability was determined by the MTT assay. Three independent experiments were performed in duplicate, and the *p*-values were calculated by using Student’s *t*-test (*** *p* < 0.001).

To further investigate the effect of *MIR452* or *siVEGFA* on VEGFR2–KRAS signaling, we assessed the expression levels of the proteins downstream of KRAS in Caco2 cells transfected with the *MIR452* mimic or *siVEGFA*. Western blotting results showed that KRAS (*p* < 0.01), BRAF (*p* < 0.05 and 0.01, respectively), and MAPK (*p* < 0.01) levels were markedly downregulated by both *MIR452* mimic and *siVEGFA* transfections in Caco2 cells (Figure 5A). We also performed the same analyses in SW48 cells. Caco2 and SW48 cells both express KRAS and BRAF [28]. The results showed that KRAS (*p* < 0.01), BRAF (*p* < 0.05), and MAPK (*p* < 0.05) levels were significantly downregulated by both *MIR452* mimic and *siVEGFA* transfections in SW48 cells (Figure 5B). These results clearly indicated that *MIR452* regulated the VEGFA-mediated VEGFR2–KRAS–BRAF signaling pathway in CRC cells.

### 2.10. MIR452 Inhibited CRC Cell Proliferation

The above results led us to explore the biological function(s) of *MIR452* in CRC cells. 3-(4,5-dimethylthiazol-2-yl)-2,5-diphenyl tetrazolium bromide (MTT) assay showed that cell viability was stably reduced by transfection of the CRC cell lines Caco2 (*p* < 0.001) and SW48 (*p* < 0.001; Figure 5C) with the *MIR452* mimic. Similar results were obtained with *VEGFA* gene silencing (*siVEGFA*) in CRC cells (*p* < 0.001; Figure 5C). These results indicated that *MIR452* suppressed proliferation of CRC cells by inhibiting VEGFA expression.

### 2.11. Effect of MIR452 on the Growth of Xenografted CRC Cells in Mice 

**Figure 6:**
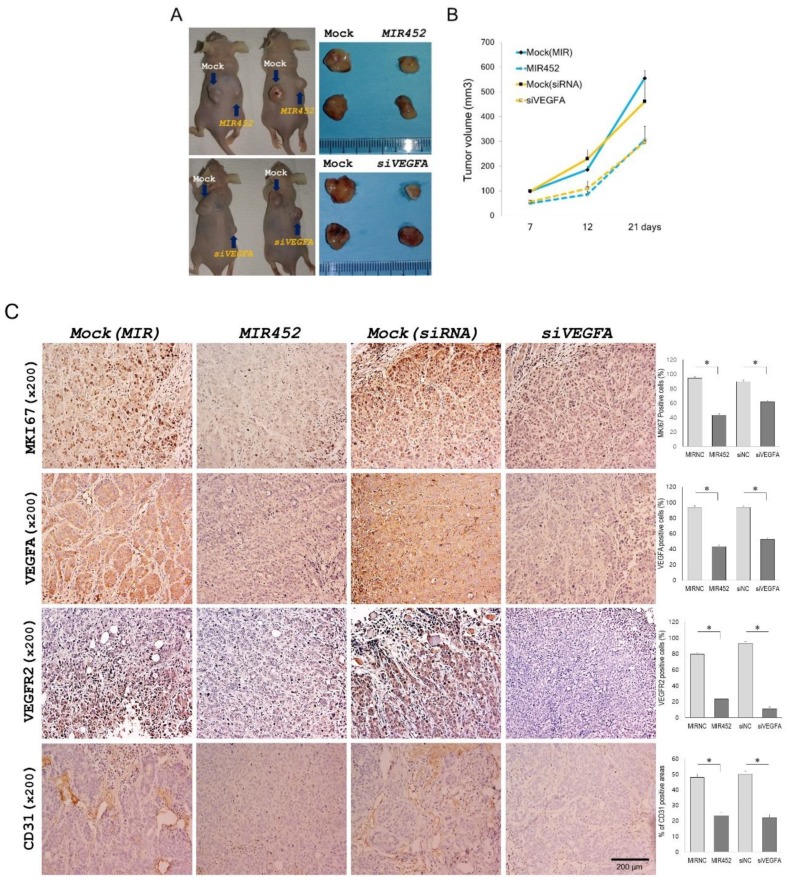
*MIR452* inhibited CRC cell growth and angiogenesis in the xenograft model. (**A**) *MIR452* inhibited CRC cell growth in vivo. An image of xenograft tumors derived from HT29 cells transfected with the *MIR452* mimic or mock control (upper panel), and the *siVEGFA* or mock control (bottom panel). (**B**) Volumes of the xenograft tumors derived from HT29 cells transfected with the *MIR452* mimic, *siVEGFA*, or mock controls in nude mice. (*n* = 8, mean ± SD). Three independent experiments were performed using 2–3 mice per experiment. (**C**) Expression of the cell proliferation marker MKI67 (the top column), VEGFA (the second column from the top), VEGFR2 (the third column from the top), and endothelial cell marker CD31 (the fourth column from the top) in the xenograft tumors formed after subcutaneous transplantation of HT29 cells transfected with the *MIR452* mimic, *siVEGFA,* or mock controls (200× magnification). Three independent experiments were performed in duplicate, and the *p*-values were calculated by using Student’s *t*-test (* *p* < 0.05).

To study the effect of *MIR452* on tumor growth in vivo, we used a xenograft tumor model consisting of athymic nude mice with subcutaneously implanted HT29 cells. After 21 days of transfection, the mean tumor volume of the *MIR452*-transfected cells was 306.1 ± 42.8 mm^3^, which was significantly smaller than that of the tumor made of the mock control cells (555.1 ± 30.7 mm^3^; Figure 6A upper panel and Figure 6B). Additionally, relative to the tumor size of the mock control cells (460.9 ± 59.1 mm^3^), the average tumor size of *siVEGFA*-transfected cells (296.8 ± 44.8 mm^3^) was significantly smaller (Figure 6A bottom panel and Figure 6B). These results indicated that the tumor cell growth was reduced by *MIR452* overexpression in CRC cells.

### 2.12. Histopathology of the Tumors Derived from the Xenografts of MIR452-Transfected CRC Cells

The xenograft tumors were immunohistochemically analyzed using antibodies against monoclonal antibodies against the proliferation marker Ki-67 (MKI67), VEGFA, VEGFR2, and CD31. The xenograft tumors derived from *MIR452* mimic- or *siVEGFA*-transfected HT29 cells contained significantly less MKI67-positive cells compared with the mock control tumors (*p* < 0.05; Figure 6C, the top column). We evaluated the VEGFA and VEGFR2 levels in the mice bearing the HT29 xenografts. *MIR452* mimic or *siVEGFA*-transfected tumors contained significantly decreased VEGFA and VEGFR2 expression compared with mock control tumors (*p* < 0.05; Figure 6C, the second and third columns from the top). We also checked the effect of *MIR452* on angiogenesis in mice bearing HT29 xenografts. Immunohistochemical staining of CD31 revealed that the blood vessel network was well developed in the mock control tumor but appeared to have been inhibited by the *MIR452* mimic or *siVEGFA* (*p* < 0.05; Figure 6C, the fourth column from the top). These results suggested that *MIR452* regulated cell proliferation and angiogenesis in the CRC cell-derived xenograft tumors by inhibiting VEGFA expression.

### 2.13. MIR452 Inhibited Angiogenesis by VEGFA Downregulation in the Rat Aortic Ring Model

**Figure 7:**
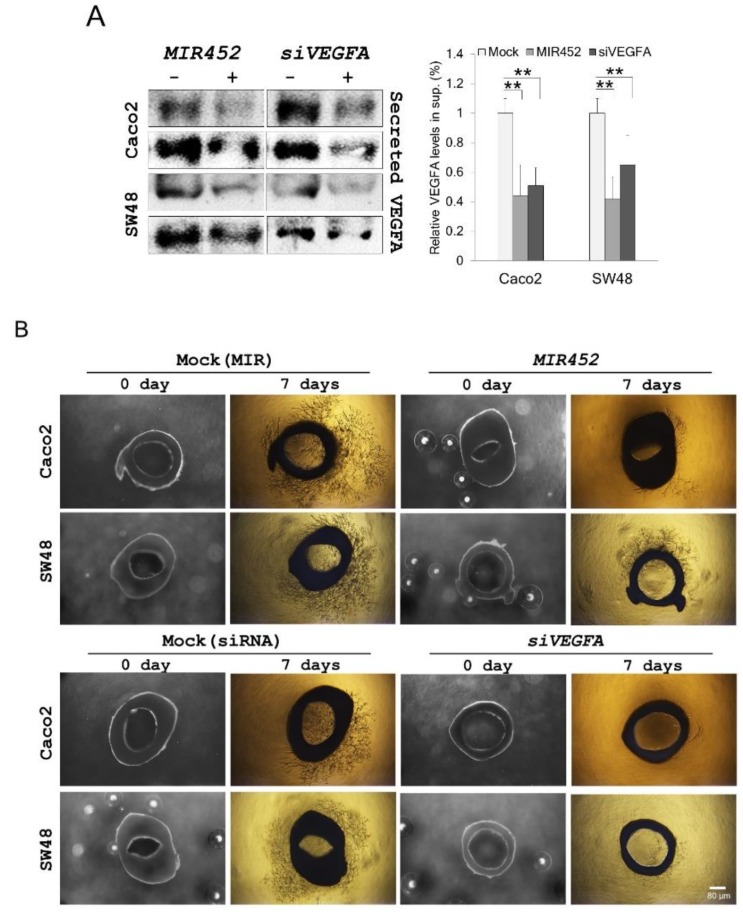
*MIR452* regulated angiogenesis by extracellular VEGFA secretion. (**A**) The extracellular VEGFA levels in Caco2 and SW48 cells transfected with the *MIR452* mimic, *siVEGFA,* or mock controls. Three independent experiments were performed in duplicate, and the *p*-values were calculated using Student’s *t*-test (** *p* < 0.01). (**B**) Rat aortic ring angiogenesis assay. The aortic rings from four-week-old Sprague–Dawley rats were randomly seeded onto Matrigel-coated wells and sealed with an overlay of Matrigel. They were co-cultured with Caco2 or SW48 cells transfected with the *MIR452* mimic (upper panel), *siVEGFA* (bottom panel), or mock controls. After 7 days, microvessel sprouting was photographed using an inverted microscope (Olympus; 2.5× magnification). Four independent experiments were performed in duplicate.

To further define the correlation between *MIR452–*VEGFA and angiogenesis, we investigated the extracellular VEGFA levels in *MIR452* mimic- or *siVEGFA*-transfected Caco2 and SW48 cells. The extracellular (secreted into the culture media) VEGFA levels were also significantly reduced by the transfection of Caco2 and SW48 cells with the *MIR452* mimic or *siVEGFA* (*p* < 0.01; Figure 7A).

On the basis of the above results, we investigated the role of VEGFA secreted from Caco2 and SW48 cells transfected with the *MIR452* mimic or *siVEGFA* in an aortic angiogenesis model. After 7 days of co-culture, the microvessel formation by *MIR452* mimic-transfected Caco2 and SW48 cells was significantly less than that observed in the mock controls (Figure 7B, upper panel). We also observed similar results with *siVEGFA*-transfected CRC cells (Figure 7B, bottom panel). These results clearly indicated that *MIR452* regulates aortic angiogenesis through VEGFA signaling.

## 3. Discussion

MiRNAs have been implicated as important post-transcriptional regulators in various biological processes as well as in the pathogenesis of various cancer types as tumor suppressor genes or oncogenes [4,8,9]. It has been well accepted that miRNAs exert their effects through their target genes. In fact, accumulating studies have recently suggested that miRNAs are essential for the tumorigenesis stages of human CRC, including cancer initiation and proliferation, apoptosis, angiogenesis, epithelial-mesenchymal transition (EMT), and cell invasion and migration [29,30]. Therefore, characterization of miRNA expression patterns and their interaction with the target genes in CRC tissues may have substantial value for disease diagnosis, prognosis, and therapy.

*MIR452* was identified as a CRC-associated miRNA by miRNA expression profiling of human CRC tissues versus healthy colorectal tissues in our previous study [26]. It was found to be upregulated in human CRC tissues compared with that in the healthy tissues [26]. To confirm this result, we increased the sample size and analyzed the *MIR452* expression levels by qRT-PCR. *MIR452* levels were mostly elevated (7 out of 10 pair) in additional CRC tissues (Figure S1A). These results indicated that *MIR452* expression may be stage-, site-, or microenvironment-specific in CRC tissues.

Twenty-seven genes were finally identified as putative target genes of *MIR452* by comparison of the mRNA microarray results with those obtained using bioinformatics algorithms (Table 1). We verified that *VEGFA* was a direct target gene of *MIR452* using dual luciferase reporter assays (Figure 1B). Furthermore, *VEGFA* mRNA and protein levels were both downregulated upon *MIR452* overexpression in CRC cells (Figure 1C) and xenograft tissues (Figure 6C). Although it is not precisely in line with our results, *MIR452* expression levels were generally increased in CRC tissues (Figure S1A). In contrast to this result, VEGFA expression levels were generally (8 out of 10 pair) downregulated in primary CRC tumor tissues compared with those in the matched healthy colon tissues (Figure 2A). Additionally, our immunohistochemistry results for the CRC tissues showed that VEGFA expression levels dramatically changed during tumorigenesis. The VEGFA expression level was downregulated at the T1 and T2 stages but upregulated at the T3 stage and then similarly expressed at the T4 stage tumor tissues relative to that in the matched healthy colon tissues (Figure 2B). These results indicate that VEGFA levels negatively correlated with *MIR452* levels in CRC tissues.

It is well known that VEGFA is associated with tumor growth, metastasis, and angiogenesis [31,32,33]. Several miRNAs have been reported to suppress tumor growth, metastasis, and angiogenesis via inhibiting the expression of their target gene *VEGFA* [34,35,36]. In this study, we found that *MIR452* suppressed CRC cell growth and migration as well as angiogenesis by inhibiting VEGFA expression. Our results showed that *MIR452* regulated the VEGFA-mediated VEGFR2 signaling pathway by directly downregulating VEGFA expression in CRC cells (Figure 3). Specifically, *MIR452* regulated the VEGFA–VEGFR2-mediated SRC, PLCG1, and MAPK signal pathways (Figure 3C, Figure 4, and Figure 5A,B). Suppression of the VEGFA-mediated VEGFR2–SRC–PTK2 signaling pathway by *MIR452* upregulation resulted in decreased cell migration and invasion (Figure 4 and Figure S2). Accumulating evidence has shown that SRC–PTK2 signaling is associated with cell migration and invasion in multiple tumor cells [37,38,39]. Furthermore, we found that the VEGFA-mediated VEGFR2–KRAS–BRAF–MAPK signaling pathway was suppressed by *MIR452* upregulation (Figure 5). These data suggest that the VEGFA–VEGFR2 signaling cascade was downregulated in CRC as a result of increased *MIR452* expression in CRC cells. This biological phenomenon involving *MIR452*-mediated VEGFA–VEGFR2 signaling was confirmed using xenograft tissues in this study (Figure 6).

Our results using xenograft mouse model showed that the expression level of the angiogenic marker CD31 was significantly decreased by *MIR452*-mediated VEGFA downregulation (Figure 6C). VEGFA mainly functioned in the extracellular space. We found that the extracellular VEGFA levels were significantly reduced in *MIR452*-overexpressing CRC cells (Figure 7A). Additionally, our rat aortic ring angiogenesis assay clearly showed that the number of microvessels formed was significantly reduced by *MIR452* mimic transfection (Figure 7B). These results strongly indicated that *MIR452* regulates angiogenesis in CRC cells by inhibiting VEGFA signaling. In actuality, several cancers including CRC are known to undergo an angiogenic switch with progression, and anti-VEGFA therapy is currently used [40,41,42]. Our results showed that MIR452 regulates cell proliferation, cell migration, and angiogenesis by direct suppressing VEGFA expression in early CRC progression; therefore, *MIR452* may have therapeutic value in relation to human CRC.

## 4. Materials and Methods

### 4.1. Patients and Tissue Samples

The tissue samples used in this study were provided by the Biobank of Wonkwang University Hospital, a member of the National Biobank of Korea. With approval from the institutional review board and informed consent from the subjects (WKIRB-201703-BR-010), we obtained 29 CRC tissues from 18 colon cancer patients (9 males and 9 females) and 11 rectal cancer patients (8 males and 3 females). The mean ages of the colon cancer patients and rectal cancer patients were 72 years and 73.6 years, respectively. The endogenous *MIR452* expression levels were assessed using 10 CRC tissue samples and matched healthy controls. In parallel, 10 CRC samples and matched healthy controls were used to evaluate VEGFA protein levels by western blotting. Additionally, a separate cohort of CRC tissue samples (7 males and 7 females) was used to assess in situ VEGFA expression by immunohistochemistry.

### 4.2. Cell Culture

The human CRC cell lines Caco2, SW480, HT29, HCT116, LoVo, and SW48 were obtained from Korean Cell Line Bank (KCLB, Seoul, Korea) or American Type Culture Collection (ATCC, Manassas, VA, USA). SW480, SW48, HCT116, LoVo, and HT29 cells were cultured at 37 °C in Roswell Park Memorial Institute Medium (RPMI) 1640 (HyClone, Logan, UT, USA), including 10% fetal bovine serum (FBS) in a humidified atmosphere of 5% CO_2_. Caco2 cells were cultured at 37 °C in a-MEM (HyClone), including 20% FBS in a humidified atmosphere of 5% CO_2_.

### 4.3. RNA Extraction and Quantitative RT-PCR

RNA extraction and quantitative RT-PCR (qRT-PCR) were carried out as we previously described [26,27,28]. The differential miRNA expression patterns were validated with qRT-PCR using a TaqMan assay (Applied Biosystems, Waltham, MA, USA), or NCode VILO miRNA cDNA Synthesis kit and EXPRESS SYBR GreenER miRNA qRT-PCR kit (Invitrogen, Carlsbad, CA, USA). The mRNA levels were assessed with qRT-PCR using SYBR Green dye (Applied Biosystems). RNU48 (for TaqMan qRT-PCR) or 5.8S (for SYBR qRT-PCR), and *GAPDH* were used as endogenous controls of miRNA and mRNA qRT-PCR, respectively. Each sample was run in triplicate. The primers used are listed in Table S2.

### 4.4. Transfection and Oligonucleotides

SW480, HCT116, SW48, and HT29 cells (3 × 10^5^) or Caco2 cells (1.5 × 10^5^) were plated on six-well culture plates or 10 cm dishes and cultured as described above. The *MIR452* mimic (hsa-miR-452, pre-miR miRNA precursor AM17100, product ID: PM12946) and negative control oligonucleotides were commercially synthesized (Ambion, Austin, TX, USA), and used at 50 nmol/mL for transfections. The transfections were performed with Lipofectamine RNAiMAX (Invitrogen) or siPORT *NeoFX* transfection agent (Ambion) according to the manufacturers’ recommendations. The *VEGFA* small interfering RNA (siRNA) and negative control siRNA transfections were performed according to the manufacturer’s protocol (Ambion). The cells were harvested 24–48 h (for miRNA and mRNA expression) or 48–72 h (for protein expression) after transfection for functional assays or RNA/protein extraction.

### 4.5. Identification of the MIR452 Target Genes by mRNA Expression Profiling 

SW480 and Caco2 cells were transfected with the *MIR452* mimic. The total RNAs were isolated 48 h after transfection. They were amplified and purified using the Illumina Total Prep RNA Amplification Kit (Ambion) according to the manufacturer’s instructions, eventually yielding biotinylated complementary RNAs (cRNAs). Hybridization of the samples, signal detection, array scanning, and data analysis and filtering were carried out as previously described [26,27,28].

### 4.6. Luciferase Reporter Assay

Wild-type or mutant fragments of *VEGFA* 3′-UTR containing the predicted binding site of *MIR452* were amplified by PCR using the primer set shown in Table S2. The luciferase assay results were analyzed as previously described [26,27,28].

### 4.7. MIR452 Target Prediction by Bioinformatics 

The miRNA targets were predicted using the computer-aided algorithms TargetScan (http://www.targetscan.org) and miRWalk (http://www.umm.uni-heidelberg.de/apps/zmf/mirwalk/index.html).

### 4.8. Antibodies and Western Blot Analysis

Protein extraction and western blot analysis were carried out as previously described [26,27,28]. The blots were then incubated overnight at 4 °C with the primary antibodies against vascular endothelial growth factor receptor 1 (VEGFR1, also known as FLT1); VEGFR2, SRC–proto-oncogene non-receptor tyrosine kinase (SRC); protein tyrosine kinase 2 (PTK2, also known as FAK1); phospholipase C gamma 1 (PLCG1); KRAS GTPase proto-oncogene (KRAS); mitogen-activated protein kinase 3/1 (MAPK3/1, also known as ERK1/2) (Cell Signaling Technology, Danvers, MA, USA); phosphatidylinositol-4,5-bisphosphate 3-kinase catalytic subunit alpha (PIK3CA, also known as PI3K); B-Raf proto-oncogene, serine/threonine kinase (BRAF); small heat shock protein family B member 1 (HSPB1) (Santa Cruz Biotechnology, Santa Cruz, CA, USA); and VEGFA (Novus Biologicals, Littleton, CO, USA). 

### 4.9. Cell Proliferation, Cell Migration, and Transwell Migration Assays

The cell proliferation (viability), cell migration, and Transwell migration (invasion) assays were carried out as previously described [26,27,28]. The experiments were repeated at least three times in duplicate.

### 4.10. Xenograft Model 

The xenograft tumor model was established in mice as we previously described [28]. Briefly, athymic male BALB/c nude mice (6 weeks old, 19–21 g) were purchased from Charles River Technology (Boston, MA, USA) through Orient Bio Inc. (Sungnam, Gyeonggi, South Korea). The *MIR452* mimic, *siVEGFA*, or mock control oligonucleotide was incubated with Lipofectamine RNAiMAX (Invitrogen, Carlsbad, CA, USA) for 15 min at the ratio of 100 nmol oligonucleotide per 10 µL Lipofectamine. Afterward, they were mixed with HT29 cells (10^7^ cells) in RPMI 1640 medium in a final volume of 200 µL for transfection. The transfected cells were then subcutaneously injected into both sides of the posterior flanks of the mice. All surgical and care procedures that were administered to the animals were in accordance with the Animal Care Committee of Wonkwang University (WKU14-47).

### 4.11. Immunohistochemical Analysis

Immunohistochemical assays were carried out as previously described [43,44]. The antibodies and dilutions used were as follows: monoclonal antibodies against the proliferation marker Ki-67 (MKI67, Thermo Fisher Scientific, Fremont, CA, USA) (1:150) and platelet endothelial cell adhesion molecule 1 (PECAM-1, also known as CD31) (1:150), mouse anti-VEGFA (Novus Biologicals, Littleton, CO, USA) (1:50), and anti-VEGFR2 (Cell Signaling Technology, Danvers, MA, USA) (1:100).

### 4.12. Rat Aortic Ring Angiogenesis Assay

Co-culture experiments were performed in 24-well plates using the Transwell system (0.4 μm pore size) (SPL, Seoul, South Korea), whereby the CRC cells grown in the lower compartments were separated from the *MIR452* mimic- or *siVEGFA*-transfected Caco2 or SW48 cells grown on the overlying filter. All the cells were cultured as monolayers. The support medium was replaced with the assay medium 24 h before starting the co-culture. The assay medium consisted of the support medium without the additives but included 1% bovine or human serum. For the co-culture experiments, Transwell chambers were inserted into the wells and the plates were incubated at 37 °C in 99% humidity under the standard incubator conditions. Angiogenesis was studied by culturing aortic rings from the thoracic aorta of four-week-old Sprague–Dawley (Orient Bio Inc., Sungnam city, South Korea) rats in a three-dimensional medium made of growth factor-reduced Matrigel (Corning, NY, USA). Briefly, the thoracic aortas were removed from the rats sacrificed by cervical dislocation and immediately transferred to a culture dish containing ice-cold serum-free RPMI 1640 or Alpha-MEM (HyClone, Marlborough, MA, USA). The peri-aortic fibro-adipose tissue was carefully removed with fine microdissecting forceps and iridectomy scissors, paying special attention not to damage the aortic wall. Approximately 15 of 1 mm long rings were sectioned per aorta and extensively rinsed by five consecutive washes with sterile Phosphate-buffered saline (PBS) at room temperature. In parallel, 150 µL of the matrigel was added onto the Transwell filter (the upper chamber in the SPL Transwell system) in each well of a 24-well plate in a biosafety cabinet. The plate was gently shaken and then incubated at 37 °C for 30 min in an ordinary humidified incubator. Afterward, one aorta ring per well was placed onto the middle of each Transwell filter. After incubating the plates for approximately 10 min back in the incubator, another 150 µL of the matrigel per well was added to cover the rings. Then, the plates were returned to the incubator for 30 min. The aorta rings became embedded in the matrigel during this period. Next, 200 µL/well Dulbecco Modified Eagle Medium (DMEM) or RPMI 1640 containing 10% fetal bovine serum (FBS) was added. The plates were incubated at 37 °C in a humidified environment for one week and examined every other day under a microscope. At least four independent experiments were performed in triplicate.

### 4.13. Statistical Analysis

Sample size was estimated using the G*power software (Version 3.1., Heinrich Heine University, Duesseldorf, Germany). Our data showed that the mean ± SD of MIR452 levels in CRC tissues was 2.2 ± 1.1-fold. In the present study, 10 CRC tissue samples were calculated for 80% power (1-β), α = 0.05, and anticipated effect size d = 1.09. Each experiment was repeated at least three times with consistent results. The data are expressed as mean ± standard deviation (SD). Differences between the groups were assessed using the GraphPad Prism 5.0 statistical software (GraphPad Software, San Diego, CA, USA) or Student’s *t*-test. Differences with *p*-values < 0.05 were considered statistically significant.

## 5. Conclusions

**Figure 8:**
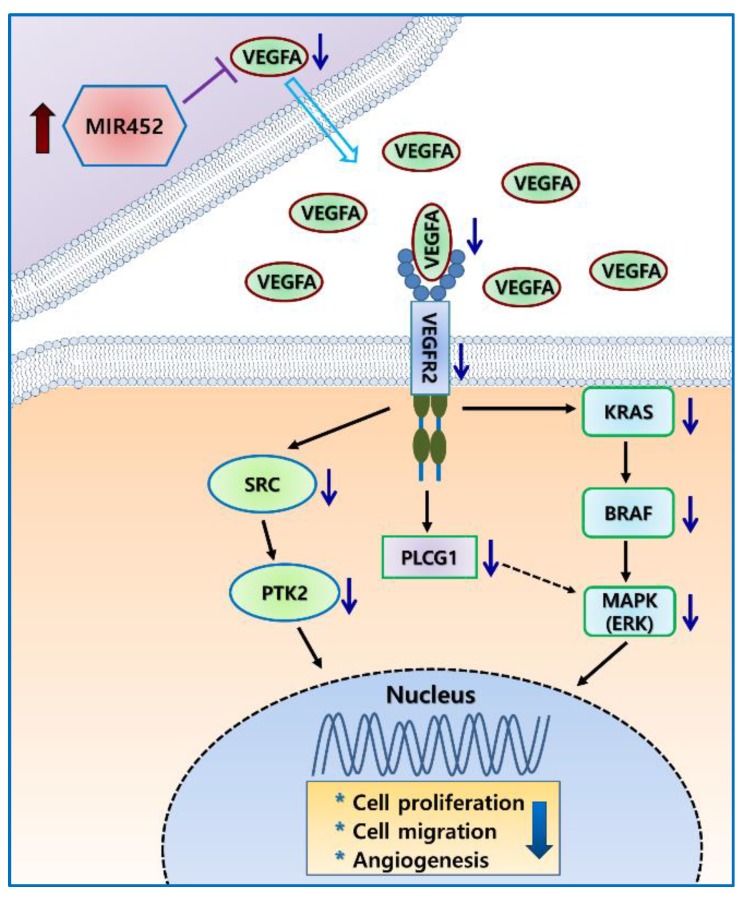
A simple putative mechanism of MIR452 regulation of VEGFA-induced cell proliferation, cell migration, and angiogenesis in human CRC. The increased MIR452 expression in CRC cells led to downregulation of cellular and extracellular VEGFA as well as VEGFR2 levels. The decreased VEGFR2 level caused inhibition of SRC, and, consequently, PTK2 was downregulated. In parallel, KRAS expression was inhibited by the downregulation of VEGFR2; this, in turn, caused inhibition of BRAF and MAPK3/1 (Extracellular signal–regulated kinase, ERK1/2) and led to the expression of growth-promoting genes. Consequently, the elevated MIR452 level in CRC led to inhibition of cell proliferation, cell migration, and angiogenesis. This simple hypothetical mechanism of MIR452-mediated inhibition of angiogenesis was based on the results of previous studies and our study presented herein.

In summary, our study found that MIR452 expression was generally upregulated in the CRC tissues. We identified 27 putative MIR452 target genes using mRNA microarray analysis of MIR452-overexpressing CRC cells and by bioinformatic tools, and showed that VEGFA was a direct target of MIR452. VEGFA expression was upregulated in the early stage CRC tissues. Moreover, MIR452 regulated two VEGFA–VEGFR2-mediated signaling pathways (VEGFR2–SRC–PTK2 and VEGFR2–KRAS–BRAF–MAPK), and as a result, MIR452 regulated cell growth, cell migration, and angiogenesis via the VEGFA–VEGFR2 pathway in CRC cells. Although we did not investigate the mechanism of MIR452 upregulation in CRC cells, our results overall suggested that the upregulated MIR452 levels during early CRC progression downregulated VEGFA expression. The diminished extracellular VEGFA levels might, in turn, downregulate VEGFR2-mediated signal pathways. Consequently, they might downregulate cell proliferation, cell migration, and angiogenesis in CRC (Figure 8). Collectively, our results suggested that downregulation of VEGFA expression by increased MIR452 levels in CRC tissues and cells might act as an early inhibitory mechanism against tumor progression in CRC tissues. Therefore, MIR452 might be a promising therapeutic target in early CRC. This possibility, however, needs to be investigated further.

## Figures and Tables

**Table 1:** The putative target genes of microRNA 452 (MIR452) identified and predicted by both the microarray analysis from the MIR452-overexpressed cells and the bioinformatics methods.

Gene Symbol	Accession	Gene Name	Chromosome Location	Functions
ARGLU1	NM_018011	arginine and glutamate rich 1	13q33.3	-
ASB8	NM_024095	ankyrin repeat and SOCS box containing 8	12q13.11	-
BCAS2	NM_005872	breast carcinoma amplified sequence 2	1p13.2	-
BTF3L4	NM_152265	basic transcription factor 3-like 4	1p32.3	-
CDK5R1	NM_003885	cyclin-dependent kinase 5, regulatory subunit 1	17q11.2	neuron-specific activator
CLK1	NM_004071	CDC-like kinase 1	2q33	protein kinases
DCUN1D1	NM_020640	DCN1, defective in cullin neddylation 1, domain containing 1	3q26.3	-
FAM134B	NM_001034850	family with sequence similarity 134, member B	5p15.1	transmembrane protein (Golgi)
FAM8A1	NM_016255	family with sequence similarity 8, member A1	6p23	-
FBXW5	NM_018998	F-box and WD repeat domain containing 5	9q34.3	ubiquitination
GTF2E1	NM_005513	general transcription factor IIE, polypeptide 1, alpha 56 kDa	3q21-q24	transcription factor
GTF2H1	NM_005316	general transcription factor IIH, polypeptide 1, 62 kDa	11p15-p14	transcription factor
IL20RA	NM_014432	interleukin 20 receptor subunit alpha	6q23.3	cytokine receptor
METTL10	NM_212554	methyltransferase like 10	10q26.13	-
MTFR1	NM_014637	mitochondrial fission regulator 1	8q13.1	mitochondrial protein
NOL8	NM_017948	nucleolar protein 8	9p22.31	-
PER2	NM_022817	period circadian clock 2	2q37.3	-
PKN2	NM_006256	protein kinase N2	1p22.2	-
PLEKHA1	NM_001001974	pleckstrin homology domain containing, family A	10q26.13	adapter protein
PPL	NM_002705	periplakin	16p13.3	desmosomes component
SHC1	NM_001130040	SHC adaptor protein 1	1q21.3	adapter protein
TAF5L	NM_001025247	TATA-box binding protein associated factor 5 like	1q42.13	histone acetylase complex
THNSL2	NM_018271	threonine synthase-like 2	2p11.2	threonine synthase
THUMPD1	NM_017736	THUMP domain containing 1	16p12.3	-
TMPRSS2	NM_005656	transmembrane protease, serine 2	21q22.3	serine protease
VEGFA	NM_001025366	vascular endothelial growth factor A	6p12	growth factor
WTAP	NM_004906	Wilms tumor 1 associated protein	6q25-q27	tumor suppressor gene

## Supplementary Materials

The following are available online at https://www.mdpi.com/2072-6694/11/10/1613/s1. Figure S1. Endogenous *MIR452* and *VEGFA* expression in CRC tissues and cell lines. Table S1. The putative target genes of MIR452 identified and predicted by the microarray analysis from the MIR452 overexpressed cells. Table S2. Primer sequences used for PCR amplification and luciferase assay in this study.
