# Supplementary material for: MicroRNA 452 Regulates Cell Proliferation, Cell Migration, and Angiogenesis in Colorectal Cancer by Suppressing VEGFA Expression
Source: Cancers (Basel). 2019 Oct 22;11(10):1613. doi: 10.3390/cancers11101613 (PMC6826374; doi:10.3390/cancers11101613)
Supplement: Supplementary file 1 [file cancers-11-01613-s001.pdf]

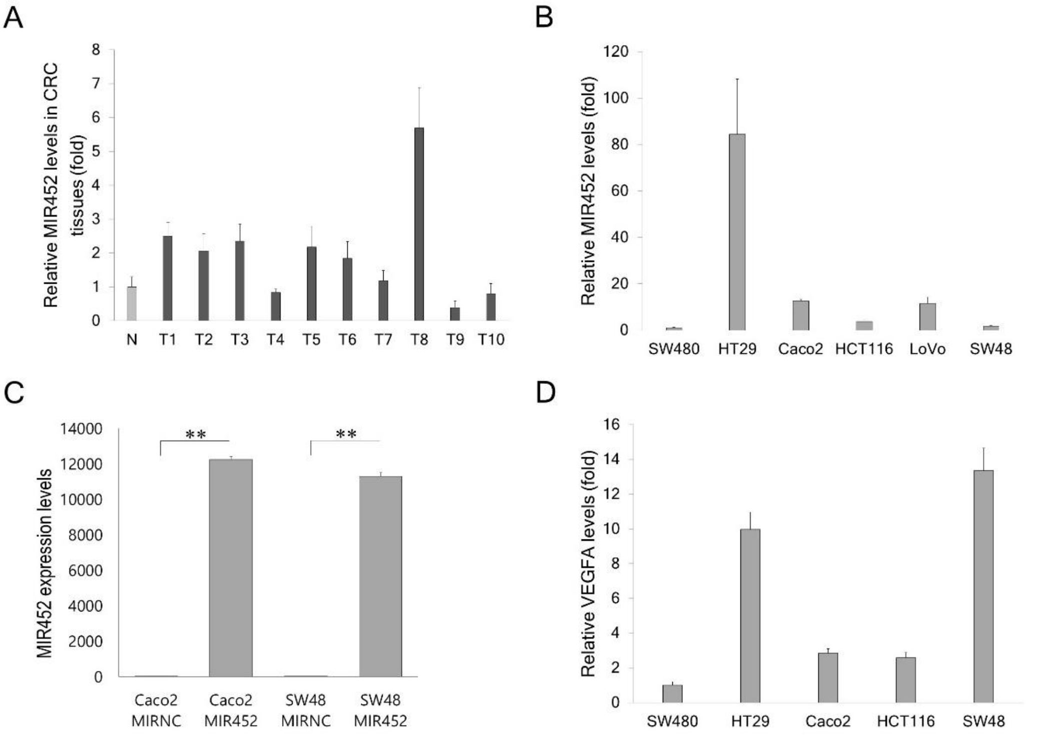

Figure S1. Endogenous MIR452 and VEGFA expression in CRC tissues and cell lines. (**A**) The expression of MIR452 was validated using 10 CRC tissue samples and matched normal colon tissue samples. miRNA levels were normalized to colon-specific RNU48. Values are presented as the fold-change in tumor tissue relative levels (ΔΔCT) to normal tissue. (**B**) The relative endogenous MIR452 expression levels in six CRC cell lines. The data are presented as a fold change in HT29, Caco2, HCT116, LoVo, and SW48 cells relative to SW480 cells. This experiment was performed as two independent experiments, each carried out in triplicate. (**C**) MIR452 expression level analysis by qRT-PCR for MIR452 transfection efficiency in Caco2 and SW48 cells. (**D**) The relative endogenous VEGFA expression levels in five CRC cell lines. The data are presented as a fold change in HT29, Caco2, HCT116, or SW48 cells relative to SW480 cells. This experiment was performed three independent experiments, each carried out in duplicate.

Table S1. The putative target genes of MIR452 identified and predicted by the microarray analysis from the MIR452 overexpressed cells.

Symbol	Definition	Accession
ACOT8	Homo sapiens acyl-CoA thioesterase 8 (ACOT8), transcript variant 1, mRNA.	NM_005469.2
ACTR6	Homo sapiens ARP6 actin-related protein 6 homolog (yeast) (ACTR6), mRNA.	NM_022496.3
ADI1	Homo sapiens acireductone dioxygenase 1 (ADI1), mRNA.	NM_018269.1
AFTPH	Homo sapiens aftiphilin (AFTPH), transcript variant 1, mRNA.	NM_203437.2
AHNAK2	Homo sapiens AHNAK nucleoprotein 2 (AHNAK2), mRNA.	NM_138420.2
AKAP7	Homo sapiens A kinase (PRKA) anchor protein 7 (AKAP7), transcript variant alpha, mRNA.	NM_004842.2
ANAPC13	Homo sapiens anaphase promoting complex subunit 13 (ANAPC13), mRNA.	NM_015391.2
ANKRD46	Homo sapiens ankyrin repeat domain 46 (ANKRD46), mRNA.	NM_198401.2

ANXA4	Homo sapiens annexin A4 (ANXA4), mRNA.	NM_001153.2
ARF4	Homo sapiens ADP-ribosylation factor 4 (ARF4), mRNA.	NM_001660.2
ARGLU1	Homo sapiens arginine and glutamate rich 1 (ARGLU1), mRNA.	NM_018011.3
ARHGEF11	Homo sapiens Rho guanine nucleotide exchange factor (GEF) 11 (ARHGEF11), transcript variant 1, mRNA.	NM_014784.2
ARL6IP1	Homo sapiens ADP-ribosylation factor-like 6 interacting protein 1 (ARL6IP1), mRNA.	NM_015161.1
ASB8	Homo sapiens ankyrin repeat and SOCS box-containing 8 (ASB8), mRNA.	NM_024095.3
ATMIN	Homo sapiens ATM interactor (ATMIN), mRNA.	NM_015251.2
ATP2B1	Homo sapiens ATPase, Ca++ transporting, plasma membrane 1 (ATP2B1), transcript variant 1, mRNA.	NM_00100132 3.1
ATP6V0A2	Homo sapiens ATPase, H+ transporting, lysosomal V0 subunit a2 (ATP6V0A2), mRNA.	NM_012463.2
B4GALT6	Homo sapiens UDP-Gal:betaGlcNAc beta 1,4-galactosyltransferase, polypeptide 6 (B4GALT6), mRNA.	NM_004775.2
BCAS2	Homo sapiens breast carcinoma amplified sequence 2 (BCAS2), mRNA.	NM_005872.2
BCLAF1	Homo sapiens BCL2-associated transcription factor 1 (BCLAF1), transcript variant 1, mRNA.	NM_014739.2
BLOC1S1	Homo sapiens biogenesis of lysosome-related organelles complex-1, subunit 1 (BLOC1S1), mRNA.	NM_001487.1
BNIP3L	Homo sapiens BCL2/adenovirus E1B 19kDa interacting protein 3-like (BNIP3L), mRNA.	NM_004331.2
BRMS1L	Homo sapiens breast cancer metastasis-suppressor 1-like (BRMS1L), mRNA.	NM_032352.3
BTF3L4	Homo sapiens basic transcription factor 3-like 4 (BTF3L4), mRNA.	NM_152265.2
BTN3A2	Homo sapiens butyrophilin, subfamily 3, member A2 (BTN3A2), mRNA.	NM_007047.3
BZW1	Homo sapiens basic leucine zipper and W2 domains 1 (BZW1), mRNA. XM_943165	NM_014670.2
C12orf48	Homo sapiens chromosome 12 open reading frame 48 (C12orf48), mRNA.	NM_017915.2
C14orf167	Homo sapiens chromosome 14 open reading frame 167 (C14orf167), transcript variant 1, non-coding RNA.	NR_023921.1
C1orf56	Homo sapiens chromosome 1 open reading frame 56 (C1orf56), mRNA.	NM_017860.3
C3orf58	Homo sapiens chromosome 3 open reading frame 58 (C3orf58), mRNA.	NM_173552.2
C4orf34	Homo sapiens chromosome 4 open reading frame 34 (C4orf34), mRNA.	NM_174921.1
C6orf62	Homo sapiens chromosome 6 open reading frame 62 (C6orf62), mRNA.	NM_030939.3
CA9	Homo sapiens carbonic anhydrase IX (CA9), mRNA.	NM_001216.1
CAMSAP1L1	Homo sapiens calmodulin regulated spectrin-associated protein 1-like 1 (CAMSAP1L1), mRNA.	NM_203459.1
CASD1	Homo sapiens CAS1 domain containing 1 (CASD1), mRNA.	NM_022900.3
CBFB	Homo sapiens core-binding factor, beta subunit (CBFB), transcript variant 2, mRNA.	NM_001755.2

CCDC61	Homo sapiens coiled-coil domain containing 61 (CCDC61), mRNA.	NM_001080402.1
CCDC90B	Homo sapiens coiled-coil domain containing 90B (CCDC90B), mRNA.	NM_021825.3
CCNG2	Homo sapiens cyclin G2 (CCNG2), mRNA.	NM_004354.1
CD164	Homo sapiens CD164 molecule, sialomucin (CD164), mRNA.	NM_006016.3
CDC40	Homo sapiens cell division cycle 40 homolog (S. cerevisiae) (CDC40), mRNA.	NM_015891.2
CDH13	Homo sapiens cadherin 13, H-cadherin (heart) (CDH13), mRNA.	NM_001257.3
CDK5R1	Homo sapiens cyclin-dependent kinase 5, regulatory subunit 1 (p35) (CDK5R1), mRNA.	NM_003885.2
CEP57	Homo sapiens centrosomal protein 57kDa (CEP57), mRNA.	NM_014679.3
CLDND1	Homo sapiens claudin domain containing 1 (CLDND1), transcript variant 1, mRNA.	NM_001040181.1
CLK1	Homo sapiens CDC-like kinase 1 (CLK1), transcript variant 2, mRNA.	NM_001024646.1
CLK4	Homo sapiens CDC-like kinase 4 (CLK4), mRNA.	NM_020666.2
CNBP	Homo sapiens CCHC-type zinc finger, nucleic acid binding protein (CNBP), mRNA.	NM_003418.1
COPG2	Homo sapiens coatomer protein complex, subunit gamma 2 (COPG2), mRNA.	NM_012133.2
COQ10B	Homo sapiens coenzyme Q10 homolog B (S. cerevisiae) (COQ10B), mRNA.	NM_025147.3
CROP	Homo sapiens cisplatin resistance-associated overexpressed protein (CROP), transcript variant 1, mRNA.	NM_016424.3
CWC22	Homo sapiens CWC22 spliceosome-associated protein homolog (S. cerevisiae) (CWC22), mRNA.	NM_020943.2
DCUN1D1	Homo sapiens DCN1, defective in cullin neddylation 1, domain containing 1 (S. cerevisiae) (DCUN1D1), mRNA.	NM_020640.2
DDX17	Homo sapiens DEAD (Asp-Glu-Ala-Asp) box polypeptide 17 (DDX17), transcript variant 2, mRNA.	NM_030881.2
DERL1	Homo sapiens Der1-like domain family, member 1 (DERL1), mRNA.	NM_024295.3
DKK3	Homo sapiens dickkopf homolog 3 (Xenopus laevis) (DKK3), transcript variant 1, mRNA.	NM_015881.5
DLEU1	Homo sapiens deleted in lymphocytic leukemia 1 (non-protein coding) (DLEU1), non-coding RNA.	NR_002605.1
DNAJC10	Homo sapiens DnaJ (Hsp40) homolog, subfamily C, member 10 (DNAJC10), mRNA.	NM_018981.1
DPM1	Homo sapiens dolichyl-phosphate mannosyltransferase polypeptide 1, catalytic subunit (DPM1), mRNA.	NM_003859.1
DPYSL2	Homo sapiens dihydropyrimidinase-like 2 (DPYSL2), mRNA.	NM_001386.4
DR1	Homo sapiens down-regulator of transcription 1, TBP-binding (negative cofactor 2) (DR1), mRNA.	NM_001938.2
ELOVL5	Homo sapiens ELOVL family member 5, elongation of long chain fatty acids (FEN1/Elo2, SUR4/Elo3-like, yeast) (ELOVL5), mRNA.	NM_021814.3
ENTPD5	Homo sapiens ectonucleoside triphosphate diphosphohydrolase 5 (ENTPD5), mRNA.	NM_001249.1

EPS8	Homo sapiens epidermal growth factor receptor pathway substrate 8 (EPS8), mRNA.	NM_004447.4
ERRFI1	Homo sapiens ERBB receptor feedback inhibitor 1 (ERRFI1), mRNA.	NM_018948.2
F2	Homo sapiens coagulation factor II (thrombin) (F2), mRNA.	NM_000506.3
FAM116A	PREDICTED: Homo sapiens family with sequence similarity 116, member A (FAM116A), mRNA.	XM_001132771.1
FAM134B	Homo sapiens family with sequence similarity 134, member B (FAM134B), transcript variant 1, mRNA.	NM_001034850.1
FAM150B	Homo sapiens family with sequence similarity 150, member B (FAM150B), mRNA.	NM_001002919.2
FAM162A	Homo sapiens family with sequence similarity 162, member A (FAM162A), mRNA.	NM_014367.3
FAM8A1	Homo sapiens family with sequence similarity 8, member A1 (FAM8A1), mRNA.	NM_016255.1
FANCI	Homo sapiens Fanconi anemia, complementation group I (FANCI), transcript variant 2, mRNA.	NM_018193.2
FBXW5	Homo sapiens F-box and WD-40 domain protein 5 (FBXW5), transcript variant 3, mRNA.	NM_178226.1
FNDC3A	Homo sapiens fibronectin type III domain containing 3A (FNDC3A), transcript variant 1, mRNA.	NM_001079673.1
FTH1	Homo sapiens ferritin, heavy polypeptide 1 (FTH1), mRNA.	NM_002032.2
FTHL8	Homo sapiens ferritin, heavy polypeptide-like 8 (FTHL8) on chromosome X.	NR_002203.1
GABPA	Homo sapiens GA binding protein transcription factor, alpha subunit 60kDa (GABPA), mRNA.	NM_002040.2
GALNS	Homo sapiens galactosamine (N-acetyl)-6-sulfate sulfatase (Morquio syndrome, mucopolysaccharidosis type IVA) (GALNS), mRNA.	NM_000512.3
GALNT1	Homo sapiens UDP-N-acetyl-alpha-D-galactosamine:polypeptide N-acetylgalactosaminyltransferase 1 (GalNAc-T1) (GALNT1), mRNA.	NM_020474.2
GDF15	Homo sapiens growth differentiation factor 15 (GDF15), mRNA.	NM_004864.1
GEN1	Homo sapiens Gen homolog 1, endonuclease (Drosophila) (GEN1), mRNA.	NM_182625.2
GGPS1	Homo sapiens geranylgeranyl diphosphate synthase 1 (GGPS1), transcript variant 2, mRNA.	NM_001037277.1
GJA3	Homo sapiens gap junction protein, alpha 3, 46kDa (GJA3), mRNA.	NM_021954.3
GNA13	Homo sapiens guanine nucleotide binding protein (G protein), alpha 13 (GNA13), mRNA.	NM_006572.3
GOLPH3L	Homo sapiens golgi phosphoprotein 3-like (GOLPH3L), mRNA.	NM_018178.3
GPER	Homo sapiens G protein-coupled estrogen receptor 1 (GPER), transcript variant 3, mRNA.	NM_001039966.1
GTF2E1	Homo sapiens general transcription factor IIE, polypeptide 1 (alpha subunit, 56kD) (GTF2E1), mRNA.	NM_005513.1
GTF2H1	Homo sapiens general transcription factor IIH, polypeptide 1, 62kDa (GTF2H1), mRNA.	NM_005316.2

HAT1	Homo sapiens histone acetyltransferase 1 (HAT1), transcript variant 1, mRNA.	NM_003642.2
HIST2H2AA3	Homo sapiens histone cluster 2, H2aa3 (HIST2H2AA3), mRNA.	NM_003516.2
HK2	Homo sapiens hexokinase 2 (HK2), mRNA.	NM_000189.4
HMGB3	Homo sapiens high-mobility group box 3 (HMGB3), mRNA.	NM_005342.2
HNRPH3	Homo sapiens heterogeneous nuclear ribonucleoprotein H3 (2H9) (HNRPH3), transcript variant 2H9, mRNA.	NM_012207.1
HSF2BP	Homo sapiens heat shock transcription factor 2 binding protein (HSF2BP), mRNA.	NM_007031.1
HSPA2	Homo sapiens heat shock 70kDa protein 2 (HSPA2), mRNA.	NM_021979.2
IGF2BP2	Homo sapiens insulin-like growth factor 2 mRNA binding protein 2 (IGF2BP2), transcript variant 1, mRNA.	NM_006548.4
IRF8	Homo sapiens interferon regulatory factor 8 (IRF8), mRNA.	NM_002163.2
ITGAV	Homo sapiens integrin, alpha V (vitronectin receptor, alpha polypeptide, antigen CD51) (ITGAV), mRNA.	NM_002210.2
ITGB3BP	Homo sapiens integrin beta 3 binding protein (beta3-endonexin) (ITGB3BP), mRNA.	NM_014288.3
JARID2	Homo sapiens jumonji, AT rich interactive domain 2 (JARID2), mRNA.	NM_004973.2
KBTBD7	Homo sapiens kelch repeat and BTB (POZ) domain containing 7 (KBTBD7), mRNA.	NM_032138.3
KIAA0261	Homo sapiens KIAA0261 (KIAA0261), mRNA.	NM_015045.1
KIAA0831	Homo sapiens KIAA0831 (KIAA0831), mRNA.	NM_014924.3
KIAA1333	Homo sapiens KIAA1333 (KIAA1333), mRNA.	NM_017769.2
KIF15	Homo sapiens kinesin family member 15 (KIF15), mRNA.	NM_020242.1
KLRG1	Homo sapiens killer cell lectin-like receptor subfamily G, member 1 (KLRG1), mRNA.	NM_005810.3
KRCC1	Homo sapiens lysine-rich coiled-coil 1 (KRCC1), mRNA.	NM_016618.1
LAMC1	Homo sapiens laminin, gamma 1 (formerly LAMB2) (LAMC1), mRNA.	NM_002293.2
LCOR	Homo sapiens ligand dependent nuclear receptor corepressor (LCOR), mRNA.	NM_032440.1
LIMCH1	Homo sapiens LIM and calponin homology domains 1 (LIMCH1), mRNA.	NM_014988.1
LIN7C	Homo sapiens lin-7 homolog C (C. elegans) (LIN7C), mRNA.	NM_018362.2
LOC100128191	PREDICTED: Homo sapiens hypothetical protein LOC100128191 (LOC100128191), mRNA.	XM_001717944.1
LOC100128309	PREDICTED: Homo sapiens hypothetical protein LOC100128309 (LOC100128309), mRNA.	XM_001717579.1
LOC100128410	PREDICTED: Homo sapiens misc_RNA (LOC100128410), miscRNA.	XR_037117.1
LOC100129882	PREDICTED: Homo sapiens similar to mCG49427 (LOC100129882), mRNA.	XM_001716882.1
LOC100130562	PREDICTED: Homo sapiens hypothetical protein LOC100130562, transcript variant 1 (LOC100130562), mRNA.	XM_001723702.1
LOC100130624	PREDICTED: Homo sapiens misc_RNA (LOC100130624), miscRNA.	XR_038453.1
LOC100131609	PREDICTED: Homo sapiens misc_RNA (LOC100131609), miscRNA.	XR_038433.1

LOC143543	Homo sapiens RNA binding motif protein, X-linked pseudogene (LOC143543) on chromosome 11.	NR_002197.1
LOC201725	Homo sapiens hypothetical protein LOC201725 (LOC201725), mRNA.	NM_00100839 3.1
LOC220433	PREDICTED: Homo sapiens similar to 40S ribosomal protein S4, X isoform (LOC220433), mRNA.	XM_941684.2
LOC388076	PREDICTED: Homo sapiens hypothetical LOC388076 (LOC388076), mRNA.	XM_001722259 .1
LOC388122	PREDICTED: Homo sapiens hypothetical LOC388122 (LOC388122), mRNA.	XM_370865.4
LOC390183	PREDICTED: Homo sapiens misc_RNA (LOC390183), miscRNA.	XR_018242.2
LOC391370	PREDICTED: Homo sapiens similar to hCG1818387 (LOC391370), mRNA.	XM_372926.5
LOC401847	PREDICTED: Homo sapiens similar to hCG1793095 (LOC401847), mRNA.	XM_001718486 .1
LOC402342	PREDICTED: Homo sapiens misc_RNA (LOC402342), miscRNA.	XR_016922.2
LOC440063	PREDICTED: Homo sapiens misc_RNA (LOC440063), miscRNA.	XR_018394.2
LOC441506	PREDICTED: Homo sapiens misc_RNA (LOC441506), miscRNA.	XR_017565.2
LOC442057	PREDICTED: Homo sapiens similar to Ciliary dynein heavy chain 11 (Axonemal beta dynein heavy chain 11) (LOC442057), mRNA.	XM_939401.1
LOC642975	PREDICTED: Homo sapiens misc_RNA (LOC642975), miscRNA.	XR_036988.1
LOC643308	PREDICTED: Homo sapiens similar to hCG1810802 (LOC643308), mRNA.	XR_018294.2
LOC644037	PREDICTED: Homo sapiens misc_RNA (LOC644037), miscRNA.	XR_017337.2
LOC645289	PREDICTED: Homo sapiens similar to CG33096-PA, isoform A (LOC645289), mRNA.	XM_928333.1
LOC645691	PREDICTED: Homo sapiens similar to heterogeneous nuclear ribonucleoprotein A1 (LOC645691), mRNA.	XM_936790.3
LOC645979	PREDICTED: Homo sapiens similar to ribosomal protein S26 (LOC645979), mRNA.	XM_001721589 .1
LOC646300	PREDICTED: Homo sapiens similar to alpha 3 type VI collagen isoform 1 precursor (LOC646300), mRNA.	XM_001130380 .1
LOC647346	PREDICTED: Homo sapiens similar to Alcohol dehydrogenase class 3 chi chain (Alcohol dehydrogenase class III chi chain) (S-(hydroxymethyl)glutathione dehydrogenase) (Glutathione-dependent formaldehyde dehydrogenase) (FDH), transcript variant 1 (LOC647346), mRNA.	XM_936495.2
LOC648210	PREDICTED: Homo sapiens similar to Heterogeneous nuclear ribonucleoprotein A1 (Helix-destabilizing protein) (Single-strand RNA-binding protein) (hnRNP core protein A1) (HDP) (LOC648210), mRNA.	XR_018923.1
LOC648638	PREDICTED: Homo sapiens similar to Peptidyl-prolyl cis-trans isomerase NIMA-interacting 4 (Rotamase Pin4) (PPIase	XM_937706.1

	Pin4) (Parvulin 14) (Par14) (Peptidyl-prolyl cis/trans isomerase EPVH) (hPar14) (LOC648638), mRNA.	
LOC648742	PREDICTED: Homo sapiens similar to growth suppressor related (LOC648742), mRNA.	XM_001714210.1
LOC653778	PREDICTED: Homo sapiens similar to solute carrier family 25, member 37 (LOC653778), mRNA.	XM_929667.1
LOC727962	PREDICTED: Homo sapiens hypothetical LOC727962 (LOC727962), mRNA.	XM_001718648.1
LOC728026	PREDICTED: Homo sapiens hypothetical LOC728026 (LOC728026), mRNA.	XM_001126659.2
LOC728115	PREDICTED: Homo sapiens misc_RNA (LOC728115), miscRNA.	XR_038319.1
LOC728467	PREDICTED: Homo sapiens misc_RNA (LOC728467), miscRNA.	XR_039554.1
LOC728481	PREDICTED: Homo sapiens similar to similar to RPL23AP7 protein (LOC728481), mRNA.	XR_015292.1
LOC728484	PREDICTED: Homo sapiens misc_RNA (LOC728484), miscRNA.	XR_015270.2
LOC728643	Homo sapiens heterogeneous nuclear ribonucleoprotein A1 pseudogene (LOC728643), non-coding RNA.	NR_003277.1
LOC728643	Homo sapiens heterogeneous nuclear ribonucleoprotein A1 pseudogene (LOC728643), non-coding RNA.	NR_003277.1
LOC728732	PREDICTED: Homo sapiens misc_RNA (LOC728732), miscRNA.	XR_038331.1
LOC729020	PREDICTED: Homo sapiens rcRPE (LOC729020), mRNA.	XM_001723111.1
LOC729423	PREDICTED: Homo sapiens similar to Heterogeneous nuclear ribonucleoprotein A1 (Helix-destabilizing protein) (Single-strand RNA-binding protein) (hnRNP core protein A1) (HDP) (LOC729423), mRNA.	XM_001726948.1
LOC729686	PREDICTED: Homo sapiens misc_RNA (LOC729686), miscRNA.	XR_039373.1
LOC729832	PREDICTED: Homo sapiens hypothetical LOC729832 (LOC729832), mRNA.	XM_001715603.1
LOC729952	PREDICTED: Homo sapiens misc_RNA (LOC729952), miscRNA.	XR_015724.2
LOC731751	PREDICTED: Homo sapiens similar to protein kinase, DNA-activated, catalytic polypeptide (LOC731751), mRNA.	XM_001129414.1
LOC732165	PREDICTED: Homo sapiens similar to Triosephosphate isomerase (TIM) (Triose-phosphate isomerase), transcript variant 2 (LOC732165), mRNA.	XM_001134259.1
LONP2	Homo sapiens lon peptidase 2, peroxisomal (LONP2), mRNA.	NM_031490.2
LYZ	Homo sapiens lysozyme (renal amyloidosis) (LYZ), mRNA.	NM_000239.1
MAP2K5	Homo sapiens mitogen-activated protein kinase kinase 5 (MAP2K5), transcript variant A, mRNA.	NM_145160.1
MAP3K1	Homo sapiens mitogen-activated protein kinase kinase kinase 1 (MAP3K1), mRNA.	NM_005921.1
MDC1	Homo sapiens mediator of DNA damage checkpoint 1 (MDC1), mRNA.	NM_014641.1
METTL10	Homo sapiens methyltransferase like 10 (METTL10), mRNA.	NM_212554.2

MFSD6	Homo sapiens major facilitator superfamily domain containing 6 (MFSD6), mRNA.	NM_017694.3
MGC10701	PREDICTED: Homo sapiens hypothetical protein MGC10701 (MGC10701), mRNA.	XM_938766.1
MGC87895	PREDICTED: Homo sapiens similar to ribosomal protein S14 (MGC87895), mRNA.	XM_942712.3
MOSPD1	Homo sapiens motile sperm domain containing 1 (MOSPD1), mRNA.	NM_019556.1
MTDH	Homo sapiens metadherin (MTDH), mRNA.	NM_178812.2
MTFR1	Homo sapiens mitochondrial fission regulator 1 (MTFR1), nuclear gene encoding mitochondrial protein, mRNA.	NM_014637.2
MX1	Homo sapiens myxovirus (influenza virus) resistance 1, interferon-inducible protein p78 (mouse) (MX1), mRNA.	NM_002462.2
MXI1	Homo sapiens MAX interactor 1 (MXI1), transcript variant 2, mRNA.	NM_130439.3
MYL7	Homo sapiens myosin, light chain 7, regulatory (MYL7), mRNA.	NM_021223.2
MYO10	Homo sapiens myosin X (MYO10), mRNA.	NM_012334.1
MYO1B	Homo sapiens myosin IB (MYO1B), mRNA.	NM_012223.2
NACAP1	Homo sapiens nascent-polypeptide-associated complex alpha polypeptide pseudogene 1 (NACAP1), non-coding RNA.	NR_002182.1
NBPF3	Homo sapiens neuroblastoma breakpoint family, member 3 (NBPF3), mRNA.	NM_032264.2
NDUFAB1	Homo sapiens NADH dehydrogenase (ubiquinone) 1, alpha/beta subcomplex, 1, 8kDa (NDUFAB1), mRNA.	NM_005003.2
NLK	Homo sapiens nemo-like kinase (NLK), mRNA.	NM_016231.4
NOC3L	Homo sapiens nucleolar complex associated 3 homolog (S. cerevisiae) (NOC3L), mRNA.	NM_022451.9
NOL8	Homo sapiens nucleolar protein 8 (NOL8), mRNA.	NM_017948.4
NUF2	Homo sapiens NUF2, NDC80 kinetochore complex component, homolog (S. cerevisiae) (NUF2), transcript variant 2, mRNA.	NM_031423.3
OAZ2	Homo sapiens ornithine decarboxylase antizyme 2 (OAZ2), mRNA.	NM_002537.2
OPTN	Homo sapiens optineurin (OPTN), transcript variant 4, mRNA.	NM_00100821 3.1
P4HA1	Homo sapiens procollagen-proline, 2-oxoglutarate 4-dioxygenase (proline 4-hydroxylase), alpha polypeptide I (P4HA1), transcript variant 1, mRNA.	NM_000917.2
PER2	Homo sapiens period homolog 2 (Drosophila) (PER2), mRNA.	NM_022817.2
PIBF1	Homo sapiens progesterone immunomodulatory binding factor 1 (PIBF1), mRNA.	NM_006346.2
PIK3CA	Homo sapiens phosphoinositide-3-kinase, catalytic, alpha polypeptide (PIK3CA), mRNA.	NM_006218.2
PJA2	Homo sapiens praja 2, RING-H2 motif containing (PJA2), mRNA.	NM_014819.3
PKN2	Homo sapiens protein kinase N2 (PKN2), mRNA.	NM_006256.2
PLA2G4D	Homo sapiens phospholipase A2, group IVD (cytosolic) (PLA2G4D), mRNA.	NM_178034.3

PLEKHA1	Homo sapiens pleckstrin homology domain containing, family A (phosphoinositide binding specific) member 1 (PLEKHA1), transcript variant 2, mRNA.	NM_001001974.1
PNRC2	Homo sapiens proline-rich nuclear receptor coactivator 2 (PNRC2), mRNA.	NM_017761.2
PPA2	Homo sapiens pyrophosphatase (inorganic) 2 (PPA2), nuclear gene encoding mitochondrial protein, transcript variant 3, mRNA.	NM_176866.2
PPAPDC2	Homo sapiens phosphatidic acid phosphatase type 2 domain containing 2 (PPAPDC2), mRNA.	NM_203453.2
PPHLN1	Homo sapiens periphilin 1 (PPHLN1), transcript variant 1, mRNA.	NM_016488.5
PPL	Homo sapiens periplakin (PPL), mRNA.	NM_002705.4
PRR15	Homo sapiens proline rich 15 (PRR15), mRNA.	NM_175887.2
PTBP2	Homo sapiens polypyrimidine tract binding protein 2 (PTBP2), mRNA.	NM_021190.1
PTGES3	Homo sapiens prostaglandin E synthase 3 (cytosolic) (PTGES3), mRNA.	NM_006601.4
PTMA	Homo sapiens prothymosin, alpha (PTMA), transcript variant 1, mRNA.	NM_001099285.1
PTP4A1	Homo sapiens protein tyrosine phosphatase type IVA, member 1 (PTP4A1), mRNA.	NM_003463.3
PURA	Homo sapiens purine-rich element binding protein A (PURA), mRNA.	NM_005859.3
QSOX1	Homo sapiens quiescin Q6 sulfhydryl oxidase 1 (QSOX1), transcript variant 2, mRNA.	NM_001004128.2
RAB6A	Homo sapiens RAB6A, member RAS oncogene family (RAB6A), transcript variant 2, mRNA.	NM_198896.1
RAD21	Homo sapiens RAD21 homolog (S. pombe) (RAD21), mRNA.	NM_006265.1
RELL1	Homo sapiens RELT-like 1 (RELL1), transcript variant 1, mRNA.	NM_001085400.1
RIMBP2	Homo sapiens RIMS binding protein 2 (RIMBP2), mRNA.	NM_015347.3
RIN2	Homo sapiens Ras and Rab interactor 2 (RIN2), mRNA.	NM_018993.2
RMI1	Homo sapiens RMI1, RecQ mediated genome instability 1, homolog (S. cerevisiae) (RMI1), mRNA.	NM_024945.2
RN5S9	Homo sapiens RNA, 5S ribosomal 9 (RN5S9), ribosomal RNA.	NR_023371.1
RNF145	Homo sapiens ring finger protein 145 (RNF145), mRNA.	NM_144726.1
RNF20	Homo sapiens ring finger protein 20 (RNF20), mRNA.	NM_019592.5
RNPEPL1	Homo sapiens arginyl aminopeptidase (aminopeptidase B)-like 1 (RNPEPL1), mRNA.	NM_018226.3
RPL14	Homo sapiens ribosomal protein L14 (RPL14), transcript variant 1, mRNA.	NM_001034996.1
RPL9	Homo sapiens ribosomal protein L9 (RPL9), transcript variant 2, mRNA.	NM_001024921.2
RSBN1	Homo sapiens round spermatid basic protein 1 (RSBN1), mRNA.	NM_018364.3
RTN3	Homo sapiens reticulon 3 (RTN3), transcript variant 1, mRNA.	NM_006054.2
S100A4	Homo sapiens S100 calcium binding protein A4 (S100A4), transcript variant 2, mRNA.	NM_019554.2

SAV1	Homo sapiens salvador homolog 1 (Drosophila) (SAV1), mRNA.	NM_021818.2
SEC24A	Homo sapiens SEC24 family, member A (S. cerevisiae) (SEC24A), mRNA.	NM_021982.1
SERINC1	Homo sapiens serine incorporator 1 (SERINC1), mRNA.	NM_020755.2
SFPQ	Homo sapiens splicing factor proline/glutamine-rich (polypyrimidine tract binding protein associated) (SFPQ), mRNA.	NM_005066.1
SFRS11	Homo sapiens splicing factor, arginine/serine-rich 11 (SFRS11), mRNA.	NM_004768.2
SKIL	Homo sapiens SKI-like oncogene (SKIL), mRNA.	NM_005414.2
SLC22A5	Homo sapiens solute carrier family 22 (organic cation transporter), member 5 (SLC22A5), mRNA.	NM_003060.2
SLC35F5	Homo sapiens solute carrier family 35, member F5 (SLC35F5), mRNA.	NM_025181.2
SLC38A2	Homo sapiens solute carrier family 38, member 2 (SLC38A2), mRNA.	NM_018976.3
SLC39A10	Homo sapiens solute carrier family 39 (zinc transporter), member 10 (SLC39A10), mRNA.	NM_020342.1
SMARCA1	Homo sapiens SWI/SNF related, matrix associated, actin dependent regulator of chromatin, subfamily a, member 1 (SMARCA1), transcript variant 1, mRNA.	NM_003069.2
SP3	Homo sapiens Sp3 transcription factor (SP3), transcript variant 2, mRNA.	NM_00101737 1.3
SPATA7	Homo sapiens spermatogenesis associated 7 (SPATA7), transcript variant 1, mRNA.	NM_018418.2
STC1	Homo sapiens stanniocalcin 1 (STC1), mRNA.	NM_003155.2
SUMO1P3	Homo sapiens SUMO1 pseudogene 3 (SUMO1P3), non-coding RNA.	NR_002190.1
TAF1D	Homo sapiens TATA box binding protein (TBP)-associated factor, RNA polymerase I, D, 41kDa (TAF1D), mRNA.	NM_024116.2
TAF5L	Homo sapiens TAF5-like RNA polymerase II, p300/CBP-associated factor (PCAF)-associated factor, 65kDa (TAF5L), transcript variant 2, mRNA.	NM_00102524 7.1
TAGLN	Homo sapiens transgelin (TAGLN), transcript variant 2, mRNA.	NM_003186.3
TATDN1	Homo sapiens TatD DNase domain containing 1 (TATDN1), mRNA.	NM_032026.2
TEF	Homo sapiens thyrotrophic embryonic factor (TEF), transcript variant 1, mRNA.	NM_003216.2
TGDS	Homo sapiens TDP-glucose 4,6-dehydratase (TGDS), mRNA.	NM_014305.2
THNSL2	Homo sapiens threonine synthase-like 2 (S. cerevisiae) (THNSL2), mRNA.	NM_018271.3
THUMPD1	Homo sapiens THUMP domain containing 1 (THUMPD1), mRNA.	NM_017736.3
THUMPD1	Homo sapiens THUMP domain containing 1 (THUMPD1), mRNA.	NM_017736.3
THUMPD1	Homo sapiens THUMP domain containing 1 (THUMPD1), mRNA.	NM_017736.3
TIMP3	Homo sapiens TIMP metallopeptidase inhibitor 3 (TIMP3), mRNA.	NM_000362.4

TMEM147	Homo sapiens transmembrane protein 147 (TMEM147), mRNA.	NM_032635.2
TMEM14B	Homo sapiens transmembrane protein 14B (TMEM14B), mRNA.	NM_030969.2
TMEM184C	Homo sapiens transmembrane protein 184C (TMEM184C), mRNA.	NM_018241.2
TMPRSS2	Homo sapiens transmembrane protease, serine 2 (TMPRSS2), mRNA.	NM_005656.2
TNNC1	Homo sapiens troponin C type 1 (slow) (TNNC1), mRNA.	NM_003280.1
TRAPPC2	Homo sapiens trafficking protein particle complex 2 (TRAPPC2), transcript variant 2, mRNA.	NM_014563.3
TRIT1	Homo sapiens tRNA isopentenyltransferase 1 (TRIT1), mRNA.	NM_017646.3
UBE2J2	Homo sapiens ubiquitin-conjugating enzyme E2, J2 (UBC6 homolog, yeast) (UBE2J2), transcript variant 1, mRNA.	NM_194315.1
UBQLN2	Homo sapiens ubiquilin 2 (UBQLN2), mRNA.	NM_013444.2
UGP2	Homo sapiens UDP-glucose pyrophosphorylase 2 (UGP2), transcript variant 1, mRNA.	NM_006759.3
VEGFA	Homo sapiens vascular endothelial growth factor A (VEGFA), transcript variant 2, mRNA.	NM_003376.4
WTAP	Homo sapiens Wilms tumor 1 associated protein (WTAP), transcript variant 1, mRNA.	NM_004906.3
YAP1	Homo sapiens Yes-associated protein 1, 65kDa (YAP1), mRNA.	NM_006106.2
YIPF4	Homo sapiens Yip1 domain family, member 4 (YIPF4), mRNA.	NM_032312.2
ZAK	Homo sapiens sterile alpha motif and leucine zipper containing kinase AZK (ZAK), transcript variant 2, mRNA.	NM_133646.2
ZNF217	Homo sapiens zinc finger protein 217 (ZNF217), mRNA.	NM_006526.2
	Homo sapiens cDNA FLJ32174 fis, clone PLACE6001064	AK056736

Table S2. Primer sequences used for PCR amplification and luciferase assay in this study.

Applications	Primers	Primer sequence (5' → 3')
qRT-PCR	VEGFA-HF	GGGCAGAATCATCACGAAGT
	VEGFA-HR	TGGTGATGTTGGACTCCTCA
	GAPDH-HF	TCACCATCTTCCAGGAGCGAGA
	GAPDH-HR	TCACTGGCATGGCCTTCCGTG
Luciferase assay	VEGFA-WF	CAGGAGCTCTCCCGAGGCACAGAGAGACA
	VEGFA-WR	CAGCTCGAGACTGTCACCGATCAGGGAG
	VEGFA-MF	AGAAATTAGGGTCTCCAATTTAATTAAAGAG
	VEGFA-MR	CTCTTTAATTAAATTGGAGACCCTAATTTCT
Taq-Man analysis	RNU48	GATGACCCCAGGTAACTCTGAGTGTGTCGC
		TGATGCCATCACCGCAGCGCTCTGACC
(FAM-NFQ)	hsa-mir-452	AACUGUUUGCAGAGGAAACUGA
